# What is the best approach to adopt for identifying the domains for a new measure of health, social care and carer-related quality of life to measure quality-adjusted life years? Application to the development of the EQ-HWB?

**DOI:** 10.1007/s10198-021-01306-z

**Published:** 2021-04-28

**Authors:** Tessa Peasgood, Clara Mukuria, Jill Carlton, Janice Connell, Nancy Devlin, Karen Jones, Rosemary Lovett, Bhash Naidoo, Stacey Rand, Juan Carlos Rejon-Parrilla, Donna Rowen, Aki Tsuchiya, John Brazier

**Affiliations:** 1 School of Health and Related Research, University of Sheffield, Sheffield, UK; 2 Personal Social Services Research Unit, University of Kent, Canterbury, UK; 3 School of Population and Global Health, University of Melbourne, Melbourne, Australia; 4 National Institute for Health and Care Excellence (NICE), London, UK; 5 Department of Economics, University of Sheffield, Sheffield, UK

**Keywords:** QALY, Extending the QALY project, PROM, Measuring and valuing health, Domain selection, Social care, Carers

## Abstract

Economic evaluation combines costs and benefits to support decision-making when assessing new interventions using preference-based measures to measure and value benefits in health or health-related quality of life. These health-focused instruments have limited ability to capture wider impacts on informal carers or outcomes in other sectors such as social care. Sector-specific instruments can be used but this is problematic when the impact of an intervention straddles different sectors.

An alternative approach is to develop a generic preference-based measure that is sufficiently broad to capture important cross-sector outcomes. We consider the options for the selection of domains for a cross-sector generic measure including how to identify domains, who should provide information on the domains and how this should be framed. Beyond domain identification, considerations of criteria and stakeholder needs are also identified.

This paper sets out the case for an approach that relies on the voice of patients, social care users and informal carers as the main source of domains and describes how the approach was operationalised in the ‘Extending the QALY’ project which developed the new measure, the EQ-HWB (EQ health and wellbeing instrument). We conclude by discussing the strengths and limitations of this approach. The new measure should be sufficiently generic to be used to consistently evaluate health and social care interventions, yet also sensitive enough to pick up important changes in quality of life in patients, social care users and carers.

## Background

Economic evaluation combines information on costs and benefits to inform the allocation of scarce resources. In the context of health care, one of the most commonly used methods is to estimate the incremental cost per quality-adjusted life year (QALY) gained. Life years gained are assigned a value on a scale anchored to one at full health and zero as equivalent to being dead. This score reflects the preferences associated with different levels of health-related quality of life (HRQoL) or health states. This value represents the ‘Q’ part of the scale and HRQoL measures such as EQ-5D, the short-form six dimensions (SF-6D) and the health utilities index (HUI 3) can be used to measure and generate this value. HRQoL measures can cover problems related to symptoms such as pain or anxiety, and functioning such as hearing or mobility but can also include the impact on activities such as personal care. These HRQoL measures have limited ability to capture the impacts of health care interventions on others who are indirect beneficiaries such as informal carers [1]. These measures are also limited to health and would not capture important outcomes in sectors such as social care, e.g. independence, confidence, safety or indeed overall wellbeing/quality of life [2–4]. Social care is the term used in the UK and some other countries to mean personal care and support to people who are disabled or severely ill.

Sector-specific preference-based measures (such as adult social care outcomes toolkit (ASCOT) [5] or ASCOT-Carer [6] for social care, and CarerQol-7D for carers [7]) offer one solution to this limitation. Within-sector QALYs can support within-sector resource allocation (although even here sector-specific outcomes may be too narrow). However, where outcomes of an intervention, such as a befriending club for the elderly, include sector-specific (e.g. increased independence) and health outcomes (e.g. reduced depression) it is not possible to combine sector-specific instruments such as ASCOT and EQ-5D. Measuring both would lead to double counting yet measuring just one would exclude potentially important benefits.

One approach to addressing this difficulty is to develop a new quality of life preference-based measure that covers all domains that are relevant and important across health and social care service users and for informal carers, anchored to a QALY scale. This would be a generic instrument, relevant to health and social care, enabling a single measure to be used across different sectors thus improving comparability of evaluations undertaken across sectors. Development of a new measure requires identification of the relevant domains or dimensions to be covered by the measure. There are choices to be made regarding how this should be done. The aim of this paper is to consider the options available for identifying domains within the context of an ongoing research project entitled ‘Extending the QALY’.^1^ .

This project is, in part, a response to growing call for a measure that captures broader well-being rather than just HRQoL and which could be accompanied by a value set to enable the generation of QALYs [8, 9]. We adopt the term ‘quality of life’ (QoL) as a placeholder for the specific theoretical judgement around what makes for a good life, and the relevant information for the purpose in which the term is being used. We are interested in ‘how good an individual’s life is’ or the ‘overall judgement of how well an individual’s life is going’ without any restriction to either subjective or objective criteria [10]. Neither the content of quality of life, nor the justification of why or how something improves quality of life, is included within this definition. However, in the context of the new measure, we are restricting the new measure to ‘health, social-care and carer-related quality of life’ rather than the whole of QoL. The concept of QoL we are interested in measuring is that which is most relevant to understanding the value for service users, and their carers, of interventions in the space of health and social care (including aged-care and services for people with an impairment or disability) and which decision makers would perceive to be relevant when evaluating the success of interventions. This includes three usually distinct concepts, health-related quality of life (HRQoL), and social care-related quality of life (SCRQoL) and carer-related QoL (the latter is sometimes referred to as care-related [7], but we opt for the term carer to avoid confusion with receiving care). This scope covers all aspects of life that could be affected by health conditions (physical and mental), health care treatments, self-management, disability, social care need or use, and the experience of caring (for family or friends).

HRQoL is not a new concept but there is still confusion on its meaning [11, 12]; some presenting it as any aspect of QoL impacted by health or health treatment others as those impacted domains of QoL which fall within the “scope of health domain” hence excluding social relationships [13]. SCRQoL is a term coined by the team which developed the ASCOT social care instrument to reflect “those aspects of QoL, or attributes, that are the focus of social care support” [14]. This construct therefore is grounded in the need for a suitable outcome to evaluate services and care provided. The third concept is carer-related QoL. This mirrors health-related QoL referring to those aspects of quality of life impacted by someone’s informal (unpaid) caring role including the impact of services provided. These concepts do not encompass every aspect of QoL, for example, political freedoms, spirituality, cultural, and aesthetic concerns.

In this paper, we consider options that are available for identifying domains for a generic measure that will be used to support decision-making in the context of health, social care and for carers. We then review the approach taken by the new EQ-HWB (health and wellbeing) instrument, finishing with a discussion of the strengths and limitations of the approach taken.

## Options for identifying domains

There are several approaches that could be taken to establish the domains of any new quality of life instrument. This includes drawing from existing measures, drawing directly from theories or asking relevant stakeholders to identify domains.

### Draw from existing HRQoL, carer-related QoL and social care-related QoL measures

A reasonable starting point for the development of a new instrument is an evaluation of what is currently available and used—and their strengths and weaknesses. This ensures that previous work is capitalised upon, including empirical work around the validity and sensitivity of current instruments and how to improve their measurement properties (for example, Longworth et al. [15]). Looking for overlap between existing measures was the approach adopted by the CarerQol-7D, which drew upon commonalities between nine key carer burden instruments [7]. Whilst this may be expedient, it faces several problems.

First, past limitations may be repeated, which includes missing out important domains or including those that are of minimal importance to service users. The research methods adopted by past instrument developers may not meet current standards since the methods of development of measures has developed significantly over the last 30 years with an increased focus on bottom–up rather than top–down approaches [16]. Second, social circumstances (such as social attitudes or the physical environment) may have changed over time resulting in the impacts of certain conditions altering with time. Third, the approach lacks theoretical rigour. Previous instruments may have different aims and objectives, and they may not have been designed to evaluate health and social care interventions. That said, understanding the domain content of existing measures could be useful as a content validity check. If the domains of the new instrument do not overlap with older instruments, we would hope that there would be a reasonable explanation as to why this might be.

The coverage and the divergence between instruments, even within a sector, is substantial. For example, the WHOQoL measure [17] and the AQoL-8D [18] are particularly different to the other HRQoL measures despite similar aims for use in the evaluation of health interventions. Table 1 in the appendix shows the domains covered within 11 well-known instruments which all aim to support economic evaluation. This disagreement in the descriptive systems of these measures creates considerable difficulty in understanding the scope of the concept of HRQoL and suggests a need for clarity on why each attribute should be considered a constituent of HRQoL and why it is sufficiently important to be included within the measure.

### Draw upon a theory of QoL

The second approach is to base instrument development around a theory of QoL. For example, CASP-19 derived its content from adopting a theoretical approach to measuring quality of life in early old age in which quality of life is “assessed as the degree to which human needs are satisfied” p. 187 [19], where those needs (control, autonomy, self-realisation and pleasure) were grounded in the theoretical work of Maslow [20], Giddens [21], Doyal and Gough [22].

There are a number of reasons why having a clear theory of quality of life that drives the development of the measure is useful: (1) any normative judgements or values implicit within the theoretical framework can be made transparent and subject to scrutiny; (2) the scope of what is to be measured is known in advance. Whether something is included as part of quality of life is a question of whether it is compatible with the chosen theory. It is, therefore, possible to be explicit and transparent about the scope of measurement; (3) theories may be amenable to empirical testing, e.g. testing the dimensionality of any model through psychometric analysis and (4) future users of the instrument are able to see its theoretical basis and judge whether this is in line with their own needs.

A clear theoretical basis is, therefore, useful both during development and for future use of an instrument. However, there are many competing theories about what makes for a good life—both in terms of what makes a life go better and why it does so. Most prominent contenders have also faced prominent criticism. Proponents of desire fulfilment or preference satisfaction accounts—in which life is improved if and only if individuals have more of the things they desire in their life—struggle (1) to adequately deal with ill-informed, meaningless or unworthy preferences, (2) to measure individual preferences beyond the proxy of goods and services, and (3) to address the role of expectations in forming desires [23]. Proponents of hedonism—in which the good life is one that has the most pleasure and the least pain [24]—struggle to address the critique that happiness is arguably not ‘the only thing that we have reason to value, nor the only metric for measuring other things that we value’ p. 26 [25]. Similarly, proponents of subjective wellbeing (SWB)—which is typically measured through life satisfaction, satisfaction with different domains of life, happiness and other positive and negative affects—struggle to address concerns that an individuals’ subjective reports of their life relate strongly to their personal expectations and frames of reference. As noted by Felce and Perry [26], these frames of reference are.

“…shaped by experience. One, cannot assume that a person's frame of reference will embrace all possibilities; it is affected by the judgment of what is possible and typical for a person in that situation.” p. 65 [26]

This paper does not attempt to give an overview of the defence against these criticisms but to simply note that they are unresolved and, as such, these approaches would be difficult to adopt as a theoretical basis to support resource allocation.

If neither mental state accounts (hedonism, or SWB) nor preference satisfaction accounts are suitable, the remaining contender would be some form of objective list account, “according to which all instances of a plurality of basic objective goods directly benefit people” p. 197 [27]. An objective list account of what makes a life go well does not provide an explanatory reason as to why something makes a life better. There is no single and common explanatory property, such as being desired, or bringing happiness or satisfaction, that justifies any attribute’s presence on the list (hence, described as a ‘plurality’ in Rice’s quote above). Each attribute on the list therefore requires additional justification [28]. The gain to the individual from basic objective goods is direct (or non-instrumental). For example, if meaningful knowledge is on the list it is because of the direct benefit to the individual of having meaningful knowledge not because of what it enables the individual to do or to feel.

Lists are derived from many different perspectives. Some are based on goods that are deduced from fundamental and moral reasons for actions [29, 30], others are based on theories of justice and entitlement [31], others from theories of the actualization of human potential [32], others on perfectionism or the development and exercise of essential human capacities [33], others on drawing together commonality across theories of psychological functioning [34], others through bringing together approaches on quality of life from different disciplines—physiology, philosophy, economics [35]. Whilst there is much overlap, there is also disagreement. For example, in the inclusion of religion and practical reasonableness [30], virtue [28], or material welfare [35]. These disagreements arise from a number of sources including: a focus on the good life or a life that is good for the individual, different opinions on human flourishing, the role of human rights within a concept of a good life, and different reasoning around whether a good can be taken as having primary or instrumental value. Finnis, below, notes commitment to removing instrumental goods from his list:

“Now besides life, knowledge, play, aesthetic experience, friendship, practical reasonableness, and religion, there are countless objectives and forms of good. But I suggest that these other objectives and forms of good will be found, on analysis, to be ways or combinations of ways of pursuing (not always sensibly) and realizing (not always successfully) one of the seven basic forms of good, or some combination of them.” p.90 [30]

Whilst it might be possible to seek some overlap between different objective list accounts, theoretical agreement is highly unlikely. Consequently, any purely theoretically driven approach may not have sufficient legitimacy to support resource allocation; it will struggle to meaningfully answer a challenge as to why one list is privileged over another. That said, a clear conceptual framework will still be useful to support the development of domains, support the structuring and collating of evidence arising from the views of future subjects (see “Draw upon the views of relevant future subjects”) and aid the understanding of relationships between domains and sub-domains. A conceptual framework can help communicate the perspective and remit of the instrument to future users and can still benefit from some of the advantages of a more explicit theoretical framework for quality of life noted above.

### Draw upon the views of relevant future subjects

The third approach to selecting the content of the new measure is to draw directly from the voice and views of stakeholders of the new instrument. Two questions arise: who to ask and how to frame the questions.

### Whom do you ask when designing a generic measure?

There are 4 potential stakeholder groups: members of the public; policy makers; experts and practitioners; and future respondents (i.e. health and social care users and carers). All members of society are potentially future users of health and social care, current and future payers of services, and beneficiaries of public health initiatives. Acknowledging overlap between groups, the average member of the public has less direct experience of health conditions, disability, social care needs, and caring than current health and social care users and carers.

The views of policy makers (e.g. HTA agencies and care commissioners), or those who may wish to use a future instrument to aid their decision-making (e.g. resource allocation decisions), are important for instrument acceptability. Policy makers are (hopefully) capable of taking a slightly detached and broader view than immediate service users who are arguably too implicated to be involved in resource allocation. A role with a responsibility for advising upon or making resource allocation decisions is likely to have encouraged considerable thought as to the relevant factors influencing decisions. Similarly, experts and practitioners in the relevant sector are likely to have given thought to their aims and what makes for a successful intervention. This experience and knowledge places them in a good position to support domain selection. The SCRQoL measure, ASCOT, for example, was based initially on a literature review of service users’ experiences and the views of policy makers and social care providers (extracted through interviews). These were then further evaluated with qualitative interviews with service users [5]. Whilst an approach that considers different perspectives is likely to generate outcomes that have practical value and acceptability to different stakeholders (i.e. policy makers, practitioners and service users), practitioners and policy makers may have opinions and life experiences that differ from the service user population. They may make different judgements about what is important to service users’ QoL. How to resolve such conflicts when determining the final content of the measure is an important issue in the development of measures used to guide and inform decision-making.

All 4 stakeholder groups are important, and ensuring the voice of current users of health and social care current informal carers particularly so, as this will help ensure that domains will be appropriate for the time (and potentially place) in which the new measure is to be used. This raises a crucial issue of how to obtain their views on what matters in their QoL.

### How do you frame the questions/discussion about quality of life?

There are four different approaches to framing questions to ask respondents about QoL to identify the themes or domains of importance:

- Ask—is X important to your life?
  One option is to identify candidate domains based on theory, existing literature, or views of experts, and then try and establish content validity through qualitative work to gather views of respondents towards these domains. Respondents may endorse or otherwise domains, but the activity will generate focusing effects. Disentangling whether an endorsement is genuine or a reluctance to challenge what is presented or the perceived wisdom of the researchers will be difficult. It may also miss aspects of QoL important to respondents.
- Ask—what matters to your life?
  Another option is to ask general questions about what is important to an individual’s life or QoL, or ‘what matters’ to them. In 2010/11 the United Kingdom’s Office for National Statistics (ONS) ran a consultation around the UK which asked, ‘what things in life matter to you?’. Similarly, within the development of the WHOQoL respondents were asked ‘what matters to your life?’ [17]. This approach has three potential problems. First, respondents may be unduly influenced by current issues, whether that be frustration with council services [37] or distrust of politicians [38]. There is a danger that the question is interpreted as ‘what is bothering you in your life now’. Respondents may not raise important domains that are not at the forefront of their mind; they may not even be aware of the value of a particular domain (such as mobility) until they face a limitation in that domain. Second, respondents may report what matters on the surface but not the underlying, more universal, concepts unless they are probed to do so. Lastly, asking broader questions about what makes for a good life may include domains that are not likely to be sensitive to change following public policy intervention, and particularly those in health and social care. There is a potential empirical solution to this—start with a broad concept of QoL and narrow the focus to those areas that are most sensitive to change following interventions based on empirical work. However, this would be time consuming and data intensive.
- Ask—‘what matters to your life’ indirectly with additional content mining
  Another option is to foster a discussion with respondents (e.g. patients or social care users) in which what matters to an individual’s QoL arises indirectly and the respondent is probed to consider the underlying values behind their views. A good example of this type of additional content mining around this question comes from the ICECAP instrument development. The interview process for the ICECAP-O is explained as:
  “In-depth interviews were informant-led, opening with broad questioning about what was important to the older people, what they enjoyed, got pleasure from, or valued in their lives.
  …. As interviews progressed, the researchers used responsive questioning to probe underlying attributes of quality of life. So, for example, if an informant said that they valued their faith, the researcher would ask them first of all for further (factual) details, covering issues such as the faith followed, what this involves and so on. The researcher would then explore with the informant what it is about faith that brings quality to their lives, asking questions such as: What is it about your faith that is important to you? How does your faith make a positive contribution to your life? What is important to you about attending worship?” [39].
  This exploratory content-mining approach [40] begins with questions that are easier to answer, e.g. How do you spend your time at moment?, and builds with the respondent a sense of what matters to them in their life through probing questions such as: “What it is about these factors that is important?”. This is cognitively less demanding for the participants and allows the interviewer to uncover underlying important aspects of what the respondent thinks makes their life go well. However, in this search for intrinsic goods (or domains) there may be a risk that a good, which has a causal relationship to another good, is treated as purely instrumental—even if it may additionally hold non-instrument value to the individual.
- Ask—how does your health condition, disability, health care, self-management, caring role, use or need for social care, impact upon your life?
  The last approach restricts the discussion to health, social care and carer-related experience. There is a clear distinction between asking broadly about an individual’s QoL versus asking about how a particular circumstance impacts upon their QoL. The interview may also use probing and less direct discussion about a respondent’s experience of their circumstances or condition to draw out underlying themes. Framing around the impact of health conditions, treatments, caring or social care use focuses the discussion onto domains that are likely to be sensitive to health and social care interventions. However, it also limits discussion to those facets in life that the respondent can cognitively attribute to a particular circumstance or condition. It may be difficult for a respondent to know whether their inability to concentrate or feel joy, for example, is a consequence of a health condition. There are also limitations due to adaptation, where service users or carers no longer experience the detrimental impacts on their QoL. It would, therefore, be important to ensure a mix of service users/carers with different experiences in terms of severity and length of time with their condition or circumstance.
  The first two approaches for framing questions are very broad and are better suited to aims where theories need to be developed rather than in the context of developing a measure with a clear focus. The latter two approaches, which are tailored towards a specific aim, are better suited towards developing a measure but will require considerations regarding which groups to include to ensure that their limitations are minimised.
  The three options for domain identification (drawing upon (i) existing measures, (ii) a theory of QoL, (iii) the views of relevant future subjects), all have strengths and limitations when considering a measure to support decision-making. Therefore, although some existing measures have used predominately one approach, using more than one of the approaches is a useful way to develop a new measure. The next section will detail the approach adopted by the extending the QALY project in the development of the EQ-HWB instrument.

## The approach adopted by the Extending the QALY project

The project aimed to develop a new instrument to measure QoL to ‘extend’ the QALY beyond HRQoL to also capture the benefits of social care and interventions on carers. The new instrument needed to be fit for the purpose of providing information to support resource allocation decisions whilst accurately reflecting the voice of the service user and carer. To achieve this, the Extending the QALY project adopted a multi-pronged approach, drawing upon the most appropriate components of the approaches discussed above (“Draw from existing HRQoL, carer-related QoL and social care-related QoL measures”, “Draw upon a theory of QoL” and “Draw upon the views of relevant future subjects”). We aimed to learn from and apply the experience of past instrument development across health, social care, carers, disability and quality of life. We also set out a clear theoretical framework for the positioning of the instrument.

### Requirements for the new measure

To meet the aim of developing a measure that is fit for purpose and reflecting the voice of the service user, the project team adopted the following requirements for the new instrument:

#### Requirement 1: covers aspects that have been identified by service users and their informal carers as important to their QoL

To be useful for resource allocation decisions, any new instrument must have broad public support and support within key subject groups (e.g. those who will be impacted by the type of decisions for which the instrument will be used, such as social care users, patients, and carers). To facilitate this role, the instrument should visibly be based on the views of service users (patients and social care users) and their informal carers and also align with common sense ideas of QoL. This should give the instrument legitimacy with the public, service users, carers, service providers and decision makers for use in supporting resource allocation.

#### Requirement 2: meets predefined criteria based on being fit for purpose

The instrument must be fit for the purpose of supporting resource allocation decisions in health care and social care. This intended use of the instrument imposes a number of constraints on domain selection, including the need to conform to good measurement properties [41, 42]. A set of criteria were drawn up for the purpose of this study [43] some of which relate to established instrument development. This included:

1. Provide a measure of QoL as a single figure within a cardinal scale anchored on zero (equivalent to dead) and one (equivalent to full quality of life) which reflects a social judgement on the value of that state
2. Be able to be treated as having interpersonal and inter-temporal comparability with a reasonable degree of confidence
3. Be inclusive and able to represent QoL for all potential future respondents (i.e. different ages (above 18 years), different genders, those in or out of work, with or without children or close family, the severely ill, those with a disability, those close to death), or, where this is not possible, be transparent about its limitations to inclusivity
4. Be amenable to routine use and inclusion in research studies and clinical trials, hence, respondents should be able to complete questions tapping into each domain with minimal burden (in terms of time, and emotional and cognitive effort)
5. Have good measurement properties including: high response rate; content validity (the process for development should be transparent and there should be broad agreement across stakeholders that all relevant outcomes are captured); face validity (there should be evidence of shared interpretation of items); be precise and able to distinguish between meaningful differences in quality of life across the full range of the scale; be responsive and sensitive to meaningful change; be reliable with high test-re-test reliability
6. Avoid double counting where possible by avoiding both instrumental and higher-level domains, and be transparent about limitations in its ability to do so
7. Be translatable into a variety of languages and ideally be relevant across cultures.

#### Requirement 3: in line with what policy makers in health and social care think is important to their decision-making

The new measure should also have broad support across policymakers and practitioners who are important stakeholders. A measure that does not meet the needs of these stakeholders would not be valuable in the context of supporting decision-making.

These three requirements ensure that the stakeholders views—both service users, other beneficiaries and service providers—are taken into account and that the measure meets good measurement properties.

### The theoretical approach adopted

The theoretical underpinning for the instrument is extra-welfarist [44] both in the commitment to a multi-dimensional measure of benefit (drawing upon an objective list account of QoL) and the role of social preferences in judging the value attributed to different states. This is in line with Culyer [45] who has argued that when evaluating healthcare in addition to utility the ‘characteristics of people’, should be taken into consideration. This incorporates the.

“…characteristics of individuals (like whether they are happy, out of pain, free to choose, physically mobile, honest). Extra-welfarism thus transcends traditional welfare: it does not exclude individual welfares from the judgements about the social state, but it does supplement them with other aspects of individuals’ p.67 [45]

The theoretical approach adopted also draws on the capabilities framework [23, 25, 46] in which the evaluative space is “people’s functioning’s (their beings and doings) and capabilities (their real or effective opportunities to achieve those functionings)” p.192 [47]. Sen recommends that the exact choice of functionings and capabilities be determined by a deliberative process with stakeholders:

“when the capability approach is used for policy work, it is the people who will be affected by the policies who should decide on what will count as valuable capabilities in this policy question” p.196 [47]

However, unless the concept of capability or the value of having the opportunity for a functioning is raised as important by carers, health and social care service users themselves and can be measured in such a way that the instrument remains fit for purpose, the focus will be on actual functioning rather than capability. Fleurbaey [48] has argued that including actual functioning in addition to capabilities is still compatible with the capabilities approach, hence, the difference in approach, particularly at the measurement level, may not be as large as it at first seems. Whilst the lack of commitment to incorporating capability or freedoms, in addition to functioning distinguishes our approach from a capabilities approach, it is still worth acknowledging that this work draws on, and benefits from, the academic lineage of the capabilities framework.

### Domain selection approach

The approach taken to select attributes of importance was to base these directly on the views of service users and carers identified through a large-scale literature review of qualitative studies. Additional qualitative work was considered unnecessary based on breadth of existing qualitative research (spanning a range of disciplines) that focuses upon health (physical and mental), social care and carer-related quality of life.

Drawing on existing literature enables coverage of many different health conditions, different types of social care users and carers across different age ranges, different ethnicities, and geographic locations (the review included literature from Europe, USA, Canada, Australia and New Zealand). It also enables the material to be drawn from many different qualitative interviewers and researchers, who may adopt different interview styles and questions, and approach their data with different assumptions and perspectives. This breadth of coverage would simply not be feasible with primary qualitative work. Indeed, the relevant, high-quality published material is so extensive that for many areas of interest, it was possible to rely upon existing qualitative reviews. The review also included qualitative work done in the development of other measures. The full details of the literature review are reported elsewhere [49].

Data extraction and synthesis applied the ‘framework’ method. This is a structured approach to organising and analysing data that begins with a conceptual framework that is modified and expanded upon as new themes emerge from the data extraction [50]. This required an a priori conceptual model to support data extraction. The structure of the initial conceptual model developed for the framework analysis was based on the widely known Wilson and Cleary’s [51] model of health-related quality of life^2^. Their model links biological and physiological variables to symptom status and then to functional health, then health perceptions and finally overall quality of life. These pathways are placed in the context of personal and environmental factors, which act as mediators.

**Fig. 1:**
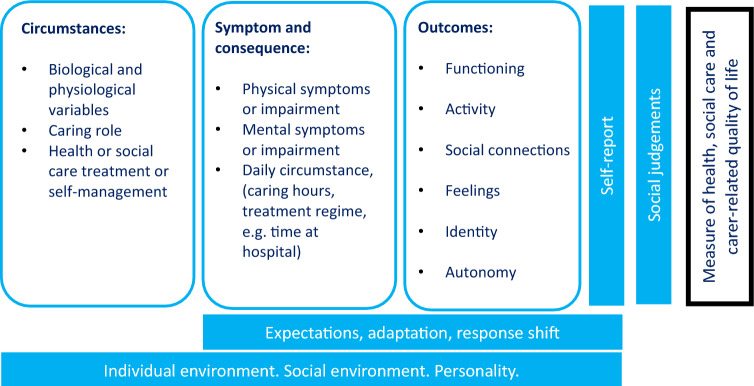
Initial conceptual model adapted from Wilson and Clearly [43]

Given the aim of moving beyond HRQoL, we amended the Wilson and Cleary model to include aspects beyond biological functioning such as being a carer or being in receipt of health or social care (Fig. 1). These circumstances may impact upon physical or mental symptoms or impairments, in addition to daily circumstances (such as time spent caring or undertaking treatment). These symptoms and circumstances were seen in turn as impacting on functioning and activity, social connectedness, feelings and physical sensations and identity.

In line with Wilson and Cleary [51], we placed the model within the context of a social and individual environmental and personality factors as mediators. Given that the instrument will be self-report we also flag the potential for expectations, adaptation and response shift [55] to influence both the evaluation the individual makes about their functioning and other outcomes, and their self-reports of that assessment. Our model does not incorporate a stage of ‘health perception’ that is included in the Wilson and Cleary model since the value of a state will be based on social judgement rather than individual perception. A social judgement will be made about how these components combine to make up the overall quality of an individual’s life. Whilst there are some dominant causal pathways that run left to right (such as from a biological variable, through to symptom, through to ability to achieve a particular area of functioning), there are also many complex bidirectional relationships and interconnections (social relationships for example may influence pain or mental health symptoms). Consequently, we have not included arrows in the conceptual model.

The conceptual model was used to develop the extraction framework with modification to better suit the data. For example, the separation of mental health symptoms from feelings and emotions was not consistent with the data, and autonomy/control, which was initially within ‘self-identity’ was found to be conceptually quite distinct. The data were synthesised and used to identify the themes/domains and sub-themes/domains that are relevant for quality of life in the context of health, social care and caring. This resulted in a large number of themes and sub-themes each of which was required to meet certain pre-agreed suitability criteria before they could be recommended for consideration for inclusion with the measure.

There were four criteria applied. First, there had to be reasonable consensus that this was an important aspect of quality of life for most people. In this case, the domain/sub-domain had to be raised as important for the quality of life of most patients, social care users and carers for the conditions or situations that were assessed in the review. Second, again pragmatically, as a self-report instrument the measure can only contain domains/sub-domains that are acceptable to respondents. Some respondents are likely to be unwilling to disclose information about sexual intimacy or suicidal ideation, for example. Domains that could be considered as judgmental, which may lead to social desirability issues, were also seen as impractical. For example, the literature review identified ‘being a burden to others’ as an important aspect of quality of life for some, however, this was not taken forward as it was considered too problematic in terms of acceptability (particularly to ask very elderly respondents). Third, domains/sub-domains were not taken forward where they were considered to be mostly instrumental, as long as their consequences could likely be captured elsewhere. The final list of domains should cover final outcomes, or the things people care about for their own sake, otherwise some attributes will be double counted and the length of the instrument will be unmanageable. The conceptual model helps in the consideration of what drives final outputs and where double counting may arise. However, instrumental versus non-instrumental is not a straightforward categorization. Domains/sub-domains can be thought of as both instrumental to something else of value (for example, pain has an impact on activity limitation) and of intrinsic value (it is just unpleasant to experience pain), hence, a judgement is required. Dexterity was dropped on the basis that the majority of the impact of loss of dexterity would be picked up in the activities that the individual was able to do and their feelings of frustration and confidence. Knowledge and information were dropped on the basis that this would be picked up in feelings, activities, sense of control and coping. In both examples, a case could be made that they also have intrinsic value. Fourth, domains/sub-domains considered to be covering very similar concepts, or where one concept could be seen as a component of another concept, these were merged. For example, a construct around ‘ability to keep things normal’ was merged with ‘coping’, the constructs ‘disclosure’ and ‘the reaction of others’ were merged with ‘stigma’.

The process of dropping and merging domains was iterative with extensive discussion. It included reflections back to the literature and to the terminology used by service users, and broad consultation across project governance and other consultation groups. These included a patient and public involvement and engagement (PPIE) group, members of the national institute for health and care excellence (NICE) staff, members of NICE Citizen’s Council^3^, the EuroQol Descriptive Systems Working Group, a project Steering Group (*n* = 12) and a large, on-line Advisory Group (*n* = 124). Staff from NICE were part of the study team, so that the perspective of policymakers was considered throughout the development of the new instrument. (Once the instrument is developed and valued, NICE will consider its performance and whether and when to recommend its use).

Final selection of domains draws upon face validity interviews and psychometric analysis of items along with further consultation with stakeholders. Quantitative assessment helps contextualise any theoretical differences between domains; something may be theoretically but not empirically distinguishable.

## Discussion

There are several key strengths of the approach adopted by the Extending the QALY project. First, the project applied best practice in instrument development in relation to explicit theoretical underpinnings, incorporation of qualitative work, the central role given to the voice of the service user along with policy makers and public perspectives, and the use of psychometric analysis. Second, the project aimed to be as transparent as possible in relation to decisions made throughout; including within the literature review, the criteria for the instrument overall and for selecting appropriate domains, and decisions made in matching potential domains to the criteria. Third, the project engaged in extensive consultation across a range of stakeholders.

The Extending the QALY approach aimed to develop a generic measure for use across different sectors but there are some limitations. Although the aim of the new instrument was to cover all relevant domains, there is a limit to what can be included in a generic measure. In addition, the valuation approaches that are available limit how many domains can be included in the classification system. Finally, the measure has been designed largely for self-completion. Proxy completion requires further consideration on what aspects an observer can meaningfully complete.

The focus on health, social care and carer-related quality of life, whilst of benefit in terms of future sensitivity to interventions, also potentially limits the instrument’s relevance for other sectors such as housing, prisoner well-being, and community interventions—all situations where health may be one of a number of important outcomes. The validity and sensitivity of the new instrument can be tested in different groups and interventions in the future. Testing this broader generic instrument against sector (e.g. carer measures, ASCOT) and age-specific instruments (such as ICECAP-O) will be important to understand the sensitivity and validity from this combined, generic approach.

The focus on service users and carers as a source of the domains may risk overlooking the voice of the public, who may have different concerns. We chose not to include public’s hypothetical judgements of how health, social care needs caring impacts would impact upon quality of life, preferring to rely upon actual lived experience. However, the valuation of the classification system will be based on the general population, therefore, the publics’ views will be incorporated at that stage.

This approach may be seen as adopting a deficit model, in which problems and difficulties arising from caring, health or disability status are the focus rather than the positive effects upon human capacity and flourishing. There are two responses to this. First, the domains/sub-domains identified in the review do capture attributes beyond problems and difficulties with basic living—such as self-worth, dignity, autonomy, happiness, hope, enjoyment of life, connectedness, and meaningful activity. Second, the aim of most interventions for which the new measure is intended to evaluate are more likely to be addressing deficits in quality of life arising through health, disability or ageing—seeking to give people the opportunity to lead a good quality of life rather than a fully flourishing life. Indeed, given that the opportunity cost of interventions evaluated using the new instrument may include additional years of life, a top anchor of a ‘good’ life is arguably more appropriate than a ‘perfect’ life.

The new measure is intended for use within economic evaluation of interventions especially where there are cross-sector outcomes or interventions which have an impact on others such as carers (e.g. changing the length of time in hospital). This raises the question of what the appropriate perspective should be in studies which use the new measure. Economic evaluations can be conducted from a ‘health payers’ perspective or a society perspective, with effects and costs limited to the perspective taken. For example, NICE guidance [56] focuses on direct health effects (regardless of whom they fall on) for health interventions but for public health and social care, this broadens out to other effects (implying a broader perspective), which means using several measures. The new measure may address the need to capture relevant effects beyond health although further validation evidence is needed to confirm whether all relevant aspects are indeed reflected.

This paper has set out the case for selecting domains for a new quality of life measure predominantly from the voice of patients, social care users and carers. The overall approach outlined here should support the development of a new measure which is (hopefully) sufficiently generic to be used to consistently evaluate health and social care interventions, yet also sufficiently sensitive to important changes in quality of life.

## Appendix

See Table 1.

**Table 1:** Domains included in commonly used instruments to measure HRQoL, SCRQoL, CarerQol, and capability well-being for older adults

	Health-related quality of life (HRQoL)						Capability well-being for older adults	Social care-related quality of life (SCRQoL)	Carer-social care-related quality of life	Carer-related quality of life (CarerQol)	
	EQ-5D	SF-6D	HUI mark 3	15D	AQoL-8D	WHOQoL	ICECAP-O	ASCOT	ASCOT-Carer	Carer experience scale	CarerQol-7D
Mobility/walking/physical functioning	√	√	√	√	√	√					
Physical health											√
Self-care/activities daily living	√	√			√	√					
Eating				√							
Elimination				√							
Household tasks					√						
Dexterity			√								
Breathing				√							
Usual activities/role limitation	√	√		√							
Pain/discomfort	√	√	√	√	√	√					
Cognition/memory/concentration/thinking			√	√		√					
Sexual activity/intimacy				√	√	√					
Vision			√	√	√						
Hearing			√	√	√						
Communication/speech			√	√	√						
Sleep				√	√	√					
Energy/vitality		√		√	√	√					
Control over daily life (or over caring)/autonomy/independence					√		√	√	√	√	
Personal cleanliness and comfort								√			
Food and drink								√			
Personal safety/security/freedom						√	√	√	√		
Occupation/work capacity/						√		√	√	√	
meaningful activity more, i.e. leisure, hobbies, voluntary work, studying											
Accommodation cleanliness and comfort								√			
Looking after yourself well (sleep/diet)									√		
Time and space to be yourself									√		
Social participation and involvement/social functioning		√				√		√	√		√
Social isolation					√						
Social exclusion					√						
Community role					√						
Intimate relationships (close relationships)					√		√				
Enjoy close relationships					√						
Family role					√						
Getting on with the care recipient										√	√
Feeling supported or having support (from family/friends and/or external organisations)						√			√	√	√
Depression or anxiety or mental health	√	√									√
Depression/sadness				√	√						
Anxious/stressed/worried				√	√						
Contentment					√						
Happiness/unhappy/sadness			√		√	√					
Pleasure					√		√				
Enthusiasm					√						
Coping					√						
Feeling a burden					√						
Worthlessness/self-esteem					√	√					
Confidence					√						
Self-harm					√						
Despair					√						
Anger					√						
Tranquillity/calm					√						
Fulfilment (including with carer role)/achievement							√			√	√
Financial						√					√
Body image and appearance						√					
Dependence on medicinal substances and medical aids						√					
Opportunities for acquiring new information and skills						√					
Transport						√					
Physical environment (pollution/noise/traffic/climate)						√					
Participation in and opportunities for recreation/leisure						√					
Home environment						√					
Health and social care: accessibility and quality						√					
Religion/spirituality/personal beliefs						√					

The tick represents our subjective judgement of whether the instrument captures that domain which based on (1) the items included in the instrument and (2) our interpretation of studies published by the instrument developers

## Footnotes

1 https://scharr.dept.shef.ac.uk/e-qaly/about-the-project/.

2 Alternative models were considered, including: the WHO framework for health and disability, Felce and Perry [26], Ferrans et al. [52] revision to Wilson and Cleary, Maslow's hierarchy of needs [32], Ryan and Deci's Self-Determination Theory [53], Cummins [54]. The Wilson and Clearly framework was chosen because it provided a simple structure for direct and indirect impacts of circumstances and physiological variables and a useful way of thinking about the connection to environmental and individual mediators, without over-imposing model complexity that would be incompatible with our main approach.

3 NICE Citizens Council is a panel of 30 members of the public that largely reflect the demographic characteristics of the UK (of which 5 attended a focus group to discuss domain selection). Council members are recruited by an independent organisation and serve for up to 3 years. They provide a public perspective on overarching moral and ethical issues that NICE takes account of when producing guidance.
